# Long-term follow-up of neuropsychological complications in neonates undergoing extracorporeal membrane oxygenation: a systematic review and meta-analysis

**DOI:** 10.1186/s12887-024-04564-x

**Published:** 2024-01-24

**Authors:** Shouliang Jiang, Ping Yan, Hua Wang, Jun Tang, Dezhi Mu

**Affiliations:** 1 Department of Pediatrics, West China Second University Hospital, Key Laboratory of Obstetric & Gynecologic and Pediatric Diseases and Birth Defects of Ministry of Education, Sichuan University, Chengdu, China; 2 Department of Gastroenterology, West China Hospital, Sichuan University, No. 37, Guo Xue Alley, Wu Hou District, Chengdu, 610041 Sichuan China

**Keywords:** Extracorporeal membrane oxygenation, Neurocognitive, Neurodevelopment, quality of life, Neuropsychology, Neonate

## Abstract

**Background:**

Extracorporeal membrane oxygenation (ECMO) has been widely used in severe neonatal diseases for approximately 50 years, while few studies have concentrated on the long-term follow-up of its neuropsychological development.

**Objective:**

To assess the long-term neuropsychological complications in children who underwent ECMO in infancy.

**Methods:**

The PubMed, Web of Science, Cochrane, and EMBASE databases were searched for retrieving studies published in the recent 10 years (until June 10, 2022). All studies were eligible that concentrated on the long-term follow-up of neuropsychological complications in neonates undergoing ECMO. Excluding animal studies, neonates with congenital craniocerebral dysplasia and studies with data from the same center performed at different times. Statistical analysis was performed using RevMan 5.3 and Stata/SE 12.0 software. A random-effects model was used to report results. The sensitivity analysis was utilized to identify sources of heterogeneity.

**Results:**

The meta-analysis of 10 studies that enrolled 1199 patients was conducted, showing the pooled morbidity of intelligence (pooled morbidity: 20.3%, 95% CI: 0.16-0.25, *I*^*2*^: 9.5%, *P*=0.33), motor activity (pooled morbidity: 10.3%, 95%CI: 0.07-0.14, *I*^*2*^: 43.5%, *P*=0.15), learning (pooled morbidity: 9.0%, 95%CI: -0.03-0.21, *I*^*2*^: 63.2%, *P*=0.10), hearing (pooled morbidity: 15.7%, 95%CI: 0.02-0.29, *I*^*2*^: 94.2%, *P*=0.00), vision (pooled morbidity: 18.5%, 95%CI: 0.12-0.25, *I*^*2*^: 0%, *P*=0.46), cognition (pooled morbidity: 26.3%, 95%CI: 0.19-0.34, *I*^*2*^: 0%, *P*=0.32), attention (pooled morbidity: 7.4%, 95%CI: 0.02-0.13, *I*^*2*^: 38.9%, *P*=0.20), speed in attention (pooled morbidity: 69.9%, 95%CI: 0.62-0.78), and accuracy in attention (pooled morbidity: 39.0%, 95%CI: 0.30-0.48) in neonates undergoing ECMO. The results of the Begg's test and sensitivity analysis indicated that the heterogeneity was originated from factors other than sample size.

**Conclusion:**

This systematic review and meta-analysis showed that neonates undergoing ECMO were associated with various neuropsychological complications. Additional randomized controlled trials (RCTs) with a larger sample size and a higher quality are needed.

**Supplementary Information:**

The online version contains supplementary material available at 10.1186/s12887-024-04564-x.

## Introduction

The extracorporeal membrane oxygenation (ECMO) has widely been used and developed in the past 50 years, it is recognized as a life saving therapy. However with increasing survival rates, the incidence of complications both short and long term have also increased [1, 2]. The long-term follow-up of neuropsychological complications in neonates undergoing ECMO is a major concern, and has attracted increasing research in this field [3]. What is clearly known is that, neonates undergoing ECMO have increased incidence of abnormal neuroimaging findings with a reported incidence between 10 -60 %, while it is still unreliable to indicate the long-term prognosis of patients with the central nervous system (CNS) complications only by imaging findings, which need long-term follow-up of neuropsychological development [4, 5].

In addition, an analysis of extracorporeal life support organization (ELSO) of neonates that received ECMO showed the acute neurological complications. However, long-term disability of those neonates was not reported, and follow-up time was mainly shorter than 5 years [6]. A number of factors contribute to the poor prognosis of ECMO-treated neonates, including confounding factors such as the severity of disease, potential developmental abnormalities, the length of time on ECMO, and the irrational use of anticoagulant and procoagulant medications during the onboarding process.

Thus, neonates, who have survived an extremely critical condition requiring ECMO, need structured neuropsychological follow-up. The complications of this serious disease may not appear immediately, while they may gradually develop after several years until adolescence, because the children’s health and development are expected to improve. Follow-up programs should concentrate on routine pediatric care, pay attention to the potential and acquired conditions of specific diseases, structured ECMO-neuropsychological care, including school performance, and parental education and support [7, 8].

Meanwhile, previous studies have shown that neuropsychological development would be significantly affected after premature puberty or major congenital abnormalities in full-term infants. It was reported that prepubertal and adolescent survivors were significantly affected in the areas of exercise, intelligence, hearing, vision, attention, and memory. However, follow-up of such patients has mainly been limited to single- or multi-center observational studies, and there is no systematic evaluation standard for these studies at present [9–11].

The present systematic review and meta-analysis aimed to assess the morbidity of long-term follow-up of neuropsychological complications in neonates receiving ECMO.

## Methods

### Search strategy and selection criteria

The literature search was performed by two expert researchers. The PubMed, Web of Science, Cochrane, and EMBASE databases were searched for retrieving studies published in the recent 10 years (until June 10, 2022). References of full-text articles were screened for identification of relevant studies. No language restriction was applied. The complete search strategy is presented in the Supplementary file. This systematic review and meta-analysis was performed in accordance with the Meta-analysis of Observational Studies in Epidemiology (MOOSE) reporting guideline [12] and the Preferred Reporting Items for Systematic Reviews and Meta-analyses (PRISMA) guidelines [13]. Two researchers independently assessed the title and abstract of retrieved studies. Significant disagreements were resolved through discussion. The full-text of potentially eligible studies was retrieved and independently assessed for eligibility.

The title and abstract of retrieved studies were initially screened, and full-text was reviewed with consideration of the following inclusion criteria: (a) randomized controlled trials (RCTs) and quasi-RCTs or observational studies; (b) neonates who received ECMO; (c) studies that reported independent outcomes for each mode of ECMO; (d) studies that concentrated on the long-term follow-up of neuropsychological complications in newborns who underwent ECMO; (e) the follow-up period was more than 3 years. The exclusion criteria were as follows: (a) case reports, reviews, conference abstracts, animal experiments, systematic reviews, meta-analyses, etc; (b) duplicated studies; (c) studies that did not include outcomes of interest; (d) follow-up studies with data from the same center performed at different times; (e) neonates with congenital craniocerebral dysplasia. The corresponding authors were contacted to request for additional data, if necessary.

### Data collection

The data were independently extracted by the two researchers from our team using a structured data extraction table. Disagreements were resolved through discussion. If necessary, it was attempted to contact the corresponding authors twice to obtain missing data or to clarify uncertainty. The data collection covered the characteristics of the study (study type, country, year of publication, number of ECMO centers, number of participants, gestational age, and weight of newborns), ECMO-related characteristics (mode and duration of ECMO), and long-term follow-up of neuropsychological complications in neonates. Complications were represented broadly as per the ELSO reporting guidelines. Authors were contacted for providing additional data, if necessary.

### Assessment of risk of bias and certainty of evidence

The quality of the included studies was assessed using the Newcastle-Ottawa Scale (NOS) score. The possibility of publication bias was examined using the Begg's test. The statistical heterogeneity was evaluated using the *I*^*2*^ statistics, the Chi-square test, and visual inspection of the forest plots. Heterogeneity refers to differences between the different studies included. When heterogeneity exists between studies, the results of the merger may be unreliable or the merger itself may be inappropriate. The guidelines have classified heterogeneity as follows: low-heterogeneity (*I*^*2*^ = 25–49%), moderate-heterogeneity (*I*^*2*^ = 50–74%), and high-heterogeneity (*I*^*2*^ ≥ 75%) [14]. The Grading of Recommendations, Assessment, Development and Evaluation (GRADE) approach was used to assess the certainty of evidence (GRADEpro app available online at: https://www.gradepro.org [date of access was June 15, 2022] [15].

### Statistical analysis

Statistical analysis was performed using RevMan 5.3 (Cochrane Collaboration organization, London, UK) and Stata/SE 12.0 (StataCorp LLC, College Station, TX, USA) software. A random-effects model was used to report results. All the statistical tests were two-sided, and *P* < 0.05 was considered statistically significant. The sensitivity analysis was utilized to identify sources of heterogeneity. Subgroup analysis was used to perform meta-analysis.

## Results

### Study characteristics

A total of 650 studies were initially identified using the literature search. After screening the title and abstract of the retrieved studies, 68 studies were selected. Then, 23 review articles and 23 duplicated publications were excluded. After performing literature search and study selection according to the eligibility criteria, full-text of 11 studies was evaluated. Because of overlapping data among the included studies, the study conducted by Madderom and Toussaint was excluded.

**Fig. 1:**
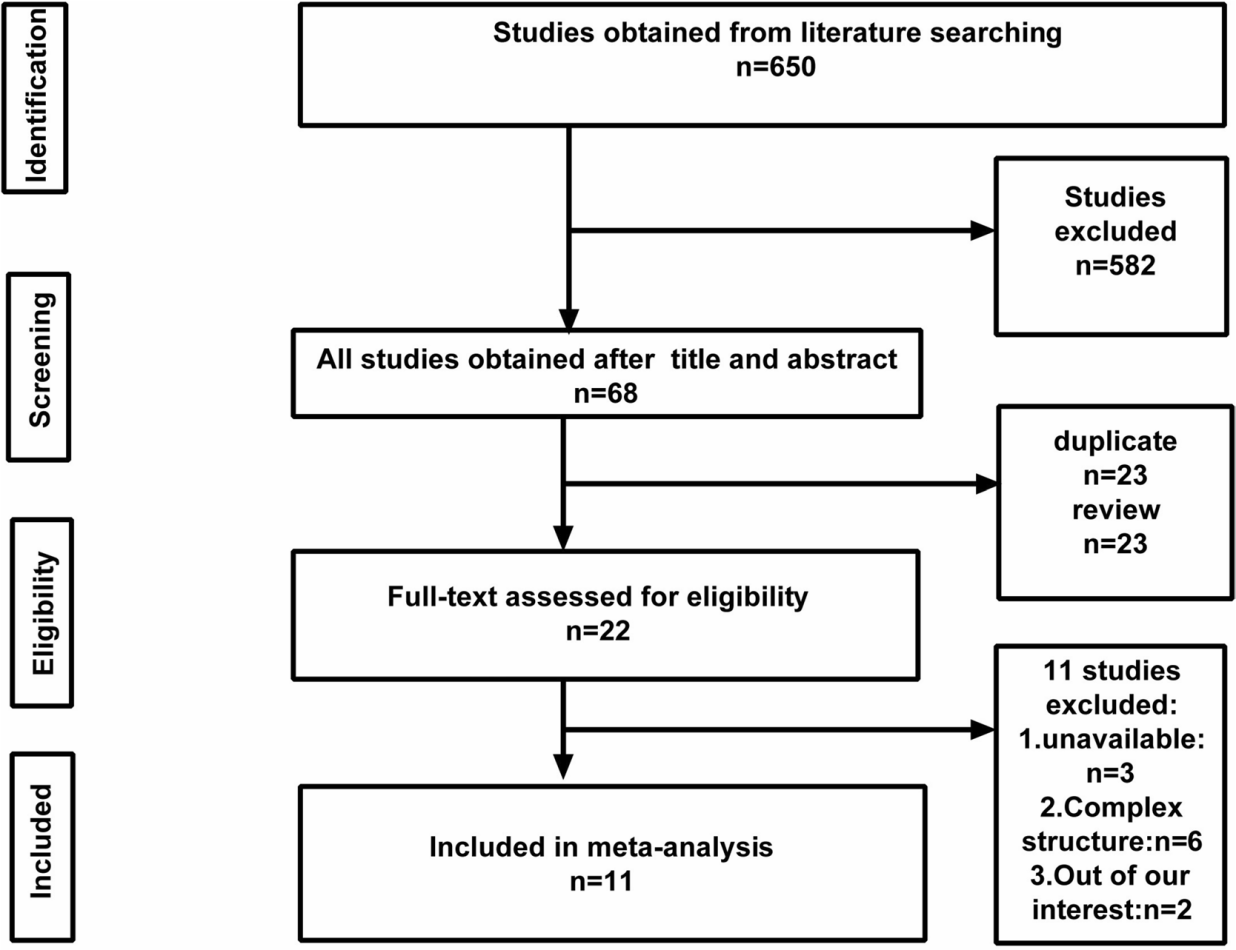
Flow chart of literature screening for the meta-analysis

Finally, 10 observational studies were included, which included 7 prospective studies, 1 cross-sectional study, 1 cohort study, and 1 retrospective study. The flowchart depicting the search strategy is shown in Fig. 1 and 1199 patients were enrolled (Table 1) [16]. The morbidity of long-term neuropsychological complications in neonates undergoing ECMO reported of each study is presented in Table 2. Moreover, 10 studies included 9 aspects of long-term neuropsychological complications in neonates undergoing ECMO, such as intelligence, learning, motor activity, hearing, vision, cognition, behavior, and attention. All these data were cross-checked and excluded from the same institution.

**Table 1:** Characteristics of the studies included in the meta-analysis.

Study(year)	Type	Countries	Centers	Number of patients					Gestational age (weeks)	Weight (kg)	VV ECMO (%)	VA ECMO (%)	VV ECMO convert to VA ECMO
				Total	CDH	MAS	PPHN	Other					
van den Hondel D(2013) [16]	Observational	Netherlands	1	136	24	73	0	39	39.6(37.8-41.4)	3.4(2.8-3.9)	0	100	0
Madderom MJ, Gischler SJ(2013) [17]	Observational	Netherlands	1	103(95)	20	46	0	29	40.0(39.0-41.0)	3.5(3.0-3.8)	NA	NA	NA
Madderom MJ, Reuser JJ(2013) [18]	Observational	Netherlands	2	135	72	30	0	33	40.0(34.0-42.0)	3.1 (2.2-4.8)	0	100	0
van der Cammen-van Zijp MH(2014) [19]	Observational	Netherlands	2	254	137	49	36	32	40.0(34.0-43.0)	3.4 (2.1-4.9)	9	90	1
Schiller RM(2016) [20]	Observational	Netherlands	2	178	97	36	0	45	40.0(38.0-42.0)	3.5(2.9-4.0)	12	87	1
Toussaint LC(2016) [21]	Observational	Netherlands	2	135	76	26	17	16	40.0(34.0-43.0)	3.4 (2.1-5.0)	3	97	0
Madderom MJ(2016) [22]	Observational	Netherlands	1	34(30)	21	5	0	8	40.0(36.0-43.0)	3.3(2.2-5.0)	0	100	0
Engle WA(2017) [23]	Observational	USA	8	146	52	23	22	49	39.0(36.7-41.3)	3.4(2.8-4.0)	27	73	0
Leeuwen L(2018) [24]	Observational	Netherlands	1	35	0	10	0	0	39.2(36.7-40.7)	3.1(2.3-3.9)	0	100	0
Reiterer F(2018) [25]	Observational	Austria	1	43	18	10	2	13	39.0(32.0-43.0)	3.4(1.8-4.2)	39	54	7

**Table 2:** Characteristics of the complications included in the meta-analysis

Study (year of publication)	Follow-up time (year)	Intelligence (%)	Learning (%)	Motor activity (%)	Hearing (%)	Vision (%)	Cognition (%)	Behavior (%)	Attention (%)
van den Hondel D (2013) [16]	5-12	0(0/136)	NA	NA	32(33/103)(CDH:21 5/24)	NA	NA	NA	NA
Madderom MJ, Gischler SJ (2013) [17]	5	NA	NA	12(10/84)	4(4/103)	0(0/102)	25(25/102)	33(31/93)	NA
Madderom MJ, Toussaint L (2013) [26]	8	38(5/14)	NA	21(3/14)	NA	NA	NA	NA	Speed:70(7/10)
									Accuracy:50(5/10)
Madderom MJ, Reuser JJ (2013) [18]	8	17(23/132)	NA	NA	NA	NA	NA	NA	Speed:70(86NA123)
									Accuracy:39(48/123)
van der Cammen-van Zijp MH (2014) [19]	5	NA	NA	14(33/243)	NA	NA	NA	NA	NA
	8			13(22/171)					
	12			43(18/42)					
Schiller RM(2016) [20]	8	22(39/178)	NA	NA	NA	NA	NA	NA	NA
Toussaint LC (2016) [21]	8	NA	NA	MAS:5(4/76)	NA	NA	NA	NA	NA
				PPHN:6(1/17)					
				CDH:19(6/26)					
				Other:13(2/16)					
Madderom MJ (2016) [22]	17-18	22(6/27)	17(5/30)	NA	NA	11(1/9)	NA	NA	NA
Engle WA (2017) [23]	≥18	NA	NA	NA	MAS:10(5/52)	MAS:14(7/52)	NA	NA	MAS:12(6/52)
					CDH:4(1/23)	CDH:22(5/23)			CDH:17(4/23)
					PPHN:23(5/22)	PPHN:23(5/22)			PPHN:9(2/22)
					Other:16(8/49)	Other:22(11/49)			Other:8(4/49)
Leeuwen L (2018) [24]	8	100(10/10)	NA	NA	NA	NA	NA	NA	NA
Reiterer F (2018) [25]	6	31(8/26)	4(1/26)	4(1/26)	NA	NA	35(9/26)	NA	4(1/26)

### Quality assessment of the included studies

The NOS score was used to perform quality assessment as all the included studies were non-RCTs. It was found that 2 studies obtained 7 stars, and 8 studies achieved 8 stars (Table 3), which demonstrated that the included studies were of high-quality. A summary of the GRADE assessment for certainty evidence is presented in Table 4, which indicated a moderate-quality for each complication.

**Table 3:** The quality assessment of all the including studies with NOS

Study (year of publication)	1	2	3	4	5	6	7	Score
van den Hondel D (2013) [16]	√	×	√	√	√	√	√	7
Madderom MJ, Gischler SJ (2013) [17]	√	√	√	√	√	√	√	8
Madderom MJ, Reuser JJ (2013) [18]	√	√	√	√	√	√	√	8
van der Cammen-van Zijp MH (2014) [19]	√	√	√	√	√	√	√	8
Schiller RM(2016) [20]	√	√	√	√	√	√	√	8
Toussaint LC (2016) [21]	√	√	√	√	√	√	√	8
Madderom MJ (2016) [22]	√	√	√	√	√	√	√	8
Engle WA (2017) [23]	√	×	√	√	√	√	√	7
Leeuwen L (2018) [24]	√	√	√	√	√	√	√	8
Reiterer F (2018) [25]	√	√	√	√	√	√	√	8

**Table 4:** The summary of the GRADE assessment for certainty evidence of each complication

**Certainty assessment**							**№ of patients**		**Effect**		**Certainty**	**Importance**
**№ of studies**	**Study design**	**Risk of bias**	**Inconsistency**	**Indirectness**	**Imprecision**	**Other considerations**	**Neurological complications**	**placebo**	**Relative (95% CI)**	**Absolute (95% CI)**		
**Pooled morbidity in intelligence (follow-up: range 5 years to ≥18 years)**												
6	observational studies	not serious	not serious	not serious	not serious	all plausible residual confounding would reduce the effect			not estimable		⨁⨁⨁◯ Moderate	CRITICAL
**Pooled morbidity in learning (follow-up: range 6 years to ≥18 years)**												
2	observational studies	not serious	not serious	not serious	not serious	all plausible residual confounding would reduce the effect			not estimable		⨁⨁⨁◯ Moderate	CRITICAL
**Pooled morbidity in motor (follow-up: range 5 years to 12 years)**												
7	observational studies	not serious	not serious	not serious	not serious	all plausible residual confounding would reduce the effect			not estimable		⨁⨁⨁◯ Moderate	CRITICAL
**Pooled morbidity in hearing (follow-up: range 5 years to ≥18 years)**												
3	observational studies	not serious	not serious	not serious	not serious	all plausible residual confounding would suggest spurious effect, while no effect was observed			not estimable		⨁⨁⨁◯ Moderate	CRITICAL
**Pooled morbidity in vision (follow-up: range 5 years to ≥18 years)**												
2	observational studies	not serious	not serious	not serious	not serious	all plausible residual confounding would reduce the effect			not estimable		⨁⨁⨁◯ Moderate	CRITICAL
**Pooled morbidity in cognitive (follow-up: mean 5.5 years)**												
2	observational studies	not serious	not serious	not serious	not serious	all plausible residual confounding would reduce the effect			not estimable		⨁⨁⨁◯ Moderate	CRITICAL
**Pooled morbidity in behavior (follow-up: mean 5 years)**												
1	observational studies	not serious	not serious	not serious	not serious	all plausible residual confounding would reduce the effect			not estimable		⨁⨁⨁◯ Moderate	CRITICAL
**Pooled morbidity in attention (follow-up: range 6 to ≥18 year)**												
4	observational studies	not serious	not serious	not serious	not serious	all plausible residual confounding would reduce the effect			not estimable		⨁⨁⨁◯ Moderate	CRITICAL
**Hearing morbidity in CDH (follow-up: range 5 to ≥18 year)**												
2	observational studies	not serious	not serious	not serious	not serious	all plausible residual confounding would reduce the effect			not estimable		⨁⨁⨁◯ Moderate	CRITICAL

### The morbidity of long-term neuropsychological complications in neonates undergoing ECMO

**Fig. 2:**
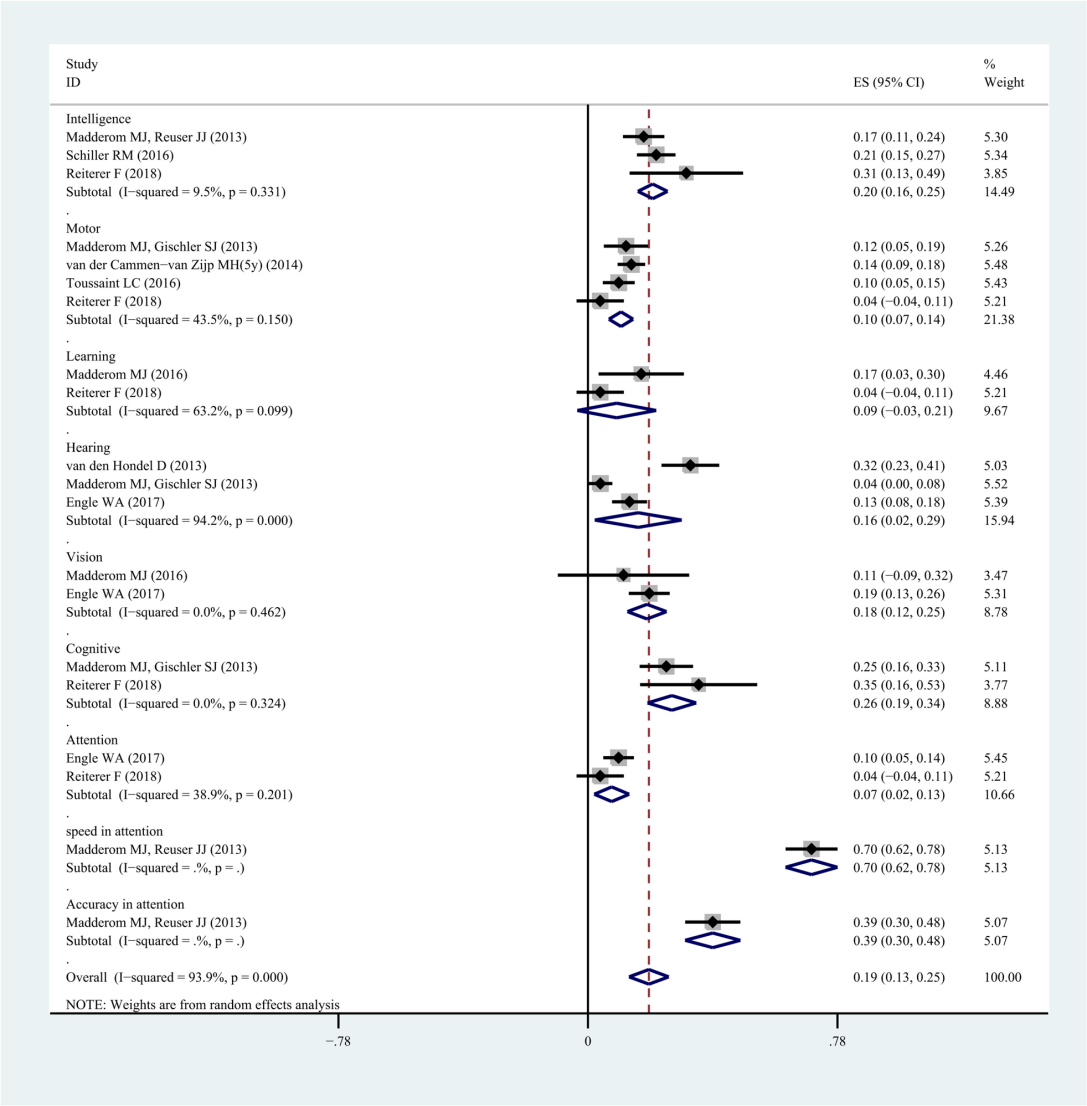
The morbidity of long-term neuropsychological developmental complications

The meta-analysis of 10 studies was performed using the subgroup analysis of complications, including 1199 patients. However, some of the data in these 10 studies are repetitive. After eliminating duplicate data, we combined the data of different institutions, which showed the pooled morbidity of intelligence (pooled morbidity: 20.3%, 95% confidence interval (CI): 0.16-0.25, *I*^*2*^: 9.5%, *P*=0.33), motor activity (pooled morbidity: 10.3%, 95%CI: 0.07-0.14, *I*^*2*^: 43.5%, *P*=0.15), learning (pooled morbidity: 9.0%, 95%CI: -0.03-0.21, *I*^*2*^: 63.2%, *P*=0.10), hearing (pooled morbidity: 15.7%, 95%CI: 0.02-0.29, *I*^*2*^: 94.2%, *P*=0.00), vision (pooled morbidity: 18.5%, 95%CI: 0.12-0.25, *I*^*2*^: 0%, *P*=0.46), cognition (pooled morbidity: 26.3%, 95%CI: 0.19-0.34, *I*^*2*^: 0%, *P*=0.32), attention (pooled morbidity: 7.4%, 95%CI: 0.02-0.13, *I*^*2*^: 38.9%, *P*=0.20), speed in attention (pooled morbidity: 69.9%, 95%CI: 0.62-0.78), and accuracy in attention (pooled morbidity: 39.0%, 95%CI: 0.30-0.48) in neonates undergoing ECMO, as illustrated in Fig. 2*.*

### The morbidity of long-term neuropsychological complications in neonates with congenital diaphragmatic hernia (CDH) undergoing ECMO

**Fig. 3:**
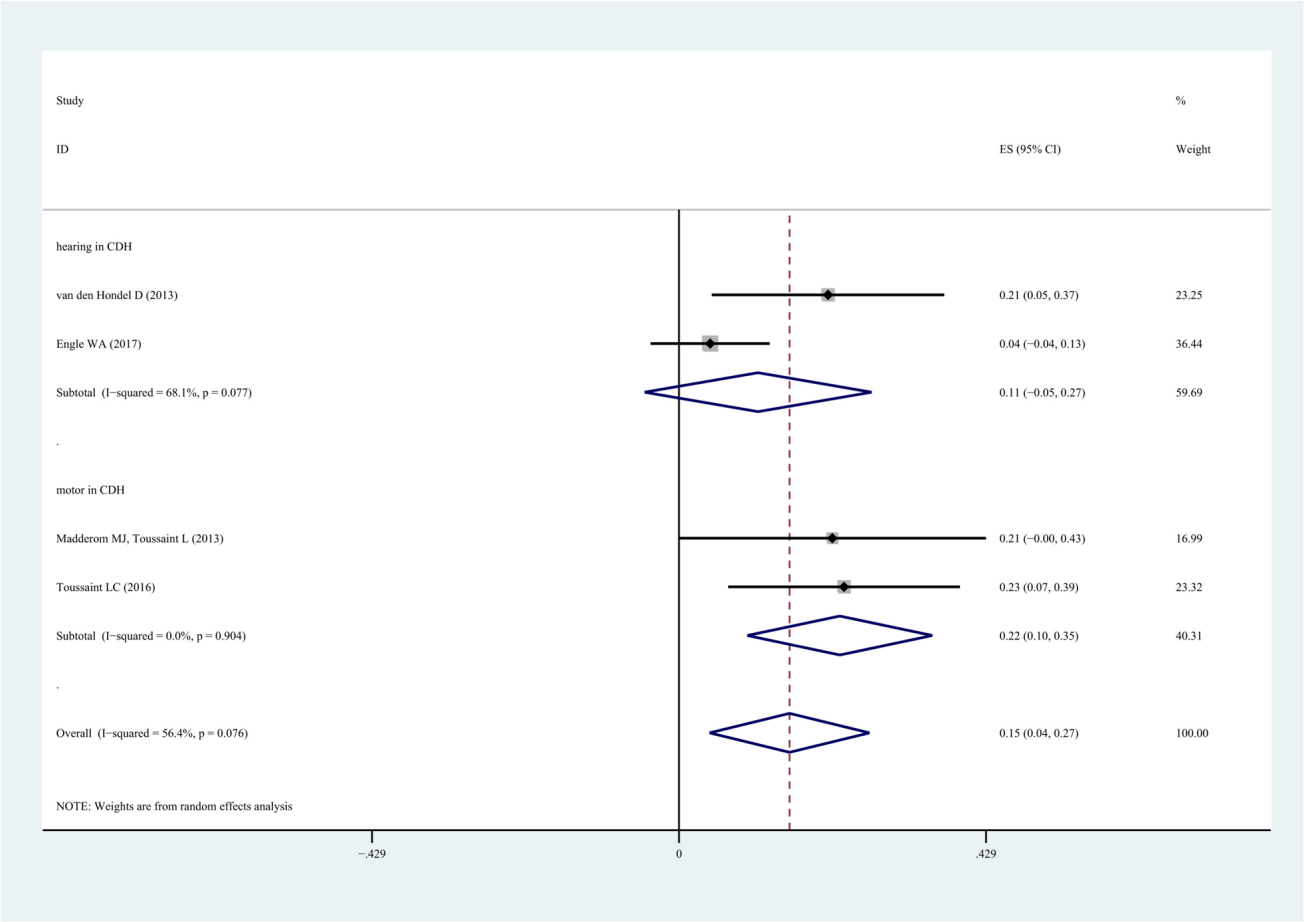
The morbidity meta-analysis of neonate CDH

A meta-analysis was conducted on the long-term neuropsychological complications of neonatal CDH undergoing ECMO reported in 4 studies using 3 subgroups of CHD, hearing, and motor activity (87 patients). The CDH subgroup showed that the pooled morbidity was 15.2% (95%CI: 0.04-0.27, *I*^*2*^: 56.4%, *P*=0.07). The pooled morbidity of hearing subgroup was 11.1% (95%CI: -0.05-0.27, *I*^*2*^: 68.1%, *P*=0.08) and the pooled morbidity of motor activity subgroup was 22.5% (95%CI: 0.10-0.35, *I*^*2*^: 0%, *P*=0.90), as shown in Fig. 3.

### The morbidity of long-term neuropsychological complications of neonatal veno-arterial-ECMO (VA-ECMO)

The morbidity of long-term neuropsychological complications of neonatal VA-ECMO reported in each study is shown in Table 5. The results revealed the pooled morbidity of intelligence (pooled morbidity: 17.4%, 95%CI: 0.11-0.24), learning (pooled morbidity: 16.7%, 95%CI: 0.03-0.30), hearing (pooled morbidity: 32.0%, 95%CI: 0.23-0.41), vision (pooled morbidity: 11.1%, 95%CI: -0.09-0.32), speed in attention (pooled morbidity: 69.9%, 95%CI: 0.62-0.78), and accuracy in attention (pooled morbidity: 39.0%, 95%CI: 0.30-0.48)*.*

**Table 5:** Characteristics of the complications included in VA-ECMO

Study (year of publication)	Follow-up time (year)	Intelligence (%)	Learning (%)	Hearing (%)	Vision (%)	Attention (%)
van den Hondel D (2013) [16]	5-12	0(0/136)	NA	32(33/103)(CDH:21 5/24)	NA	NA
Madderom MJ, Reuser JJ (2013) [18]	8	17(23/132)	NA	NA	NA	Speed:70(86/123)
						Accuracy:39(48/123)
Madderom MJ (2016) [22]	17-18	22(6/27)	17(5/30)	NA	11(1/9)	NA
Leeuwen L (2018) [24]	8	100(10/10)	NA	NA	NA	NA

### Subgroup analysis of motor activity at different follow-up periods

**Fig. 4:**
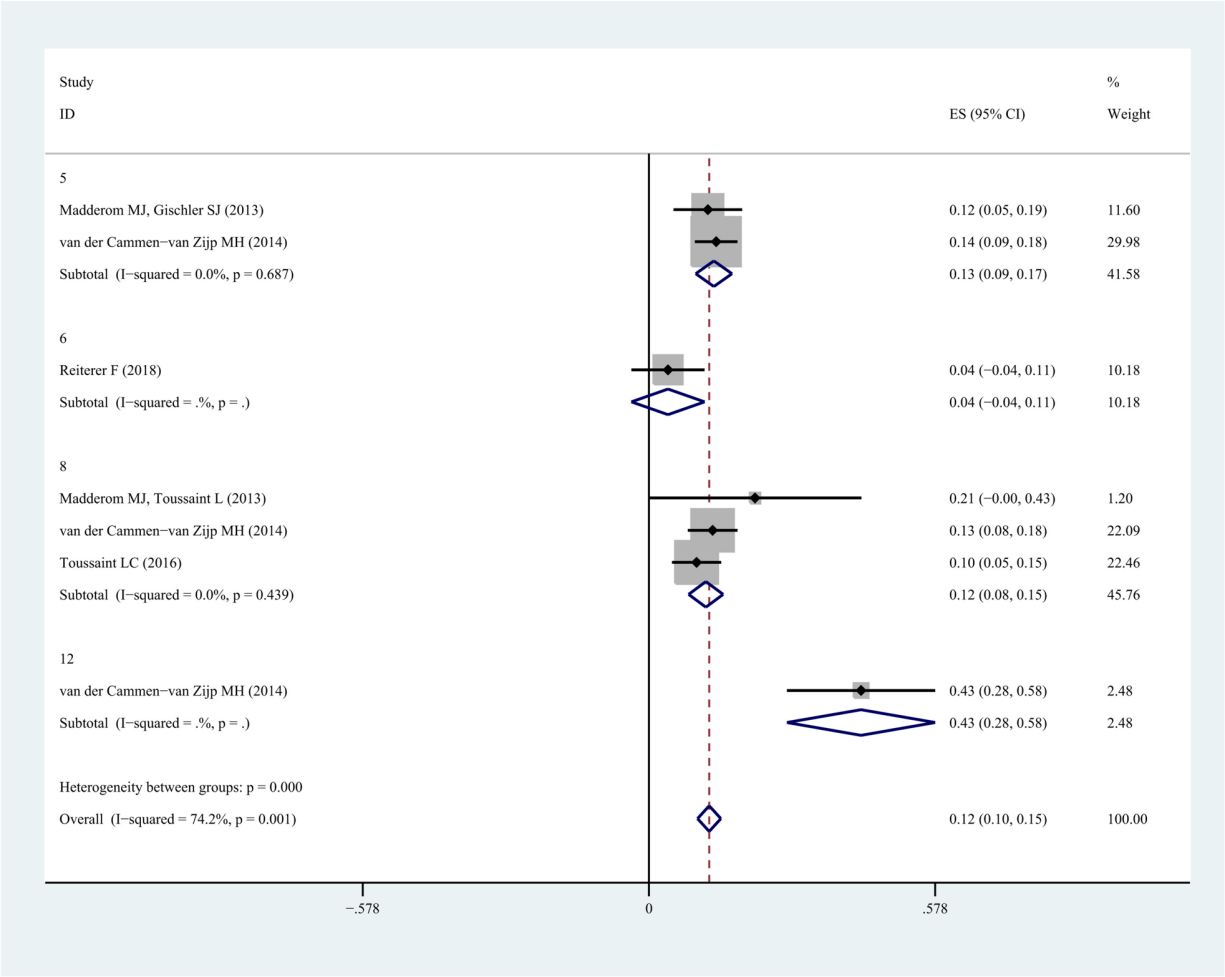
Subgroup analysis of motor activity at different follow-up periods

Subgroup analysis of motor activity was conducted at different follow-up periods. The subgroup analysis of motor activity showed that the pooled morbidity of 5 years, including 2 studies, was 13.0% (95%CI: 0.09-0.17, *I*^*2*^: 0.0%, *P*=0.17). The pooled morbidity of 6 years, including 1 study, was 4.0% (95%CI: -0.04-0.11). The pooled morbidity of 8 years, including 3 studies, was 12.0% (95%CI: 0.08-0.15, *I*^*2*^: 0.0%, *P*=0.44). Besides, the pooled morbidity of 12 years, including 1 study, was 43.0% (95%CI: 0.28-0.58), as illustrated in Fig. 4. After excluding the single-study with the high-heterogeneity, it was found that the morbidity of motor activity at 5 and 8 years was similar.

### Heterogeneity assessment

The results showed that the pooled heterogeneity of complications in learning (*I*^*2*^: 63.2%) and hearing (*I*^*2*^: 94.2%) was high (> 50%). In order to find out the source of heterogeneity in hearing, the Begg's test and sensitivity analysis were performed. The Begg's test showed *p*=0.76 > 0.05, which demonstrated that there was no publications bias. Sensitivity analysis indicated that the results were stable (risk difference (RD): 0.16, 95%CI: 0.02-0.29), which suggested that heterogeneity was originated from factors other than sample size.

## Discussion

In this systematic review and meta-analysis, we identified 10 studies (1199 patients) that described the long-term neuropsychological complications in neonates undergoing ECMO. The evidence mainly consisted of observational studies with a heterogeneous study design to assess neuropsychological development. Meta-analysis of the 10 studies (1199 patients) included 9 aspects of long-term neuropsychological complications in neonates undergoing ECMO. Reports on outcomes other than morbidity were scarce and inadequate for meta-analysis.

Of the included studies, 80% (8/10) were from the Netherlands where there were several high-volume ECMO centers and a sounder follow-up system [27, 28] and , followed by Australia and the United States accounting for 10% (1/10) of the total rate, respectively. There were 5 (50%, 5/10) single-center studies, and the remaining (50%, 5/10) were multi-center studies. Although most of the studies were from the same country, there was no overlap in data from each of our studies.

In order to evaluate the neuropsychological complications in neonates undergoing ECMO, different institutions use different assessment methods, including the Pediatric Quality of Life Inventory (PedsQL), the Movement Assessment Battery for Children (MABC), the Beery-Buktenica Developmental Test of Visual-Motor Integration (Beery VMI), the Wechsler Intelligence Scale for Children (WISC-III-NL), Subtest Digit Span of the Wechsler Adult Intelligence Scale, the Revised Amsterdam Intelligence Test (RAKIT), the Bourdon-Vos test, the Bayley Scales of Infant and Toddler Development, the Child Behavior Checklist (CBCL), the Trail Making Test, the Stroop Color-Word test, the subtests Rebus Learning and Auditory Comprehension of the Kaufman Intelligence Test, the Rey Auditory Verbal Learning Test (RAVLT), and the Rey Complex Figure Test (RCFT). Most of the evaluation methods are generally recognized, and they are mainly used in the assessment of children's neuropsychological growth and development. Although the scoring rules of different scales are different, the main contents of their evaluation are consistent, including motor, language and social adaptability. These can be reflected in the nine items studied in our study. Some of them have been rarely reported and are even subjective, inevitably leading to unreliable results were excluded [16–29].

As mentioned above, long-term neuropsychological complications of neonates undergoing ECMO included intelligence, learning, motor activity, hearing, vision, cognition, behavior, and attention. These complications have serious effects on children's neuropsychological development and even on their quality of life [30]. Moreover, neuropsychological complications become more severe over time. These neuropsychological complications are a burden not only for the individual but even for society. For such children, we need to take more careful care until they become adults, which requires more investment in money and time. Even as adults, we still need to give some people special care. However, these efforts are worth it, because these people will also create different values for the society. More importantly, through the ECMO technology, more people have the chance to survive, so that they have the right to life.

The morbidity rates of different complications were statistically determined in the present study. Although a series of analyses were performed on neuropsychological complications in neonate undergoing ECMO in the included studies, some of studies did not provide insufficient evidences, which led to difficulties to find the reasons in data analysis, and restricted the generalization of more detailed results. Studies on the neuropsychological complications of neonatal CDH in neonate undergoing ECMO accounted for the majority. The pooled morbidity of hearing in neonatal CDH was 11.1% (95%CI: -0.05-0.27, *I*^*2*^: 68.1%, *P*=0.08) and the pooled morbidity of motor activity in neonatal CDH was 22.5% (95%CI: 0.10-0.35, *I*^*2*^: 0%, *P*=0.90). Meanwhile, we could summarize the morbidity of complications of VA-ECMO and the morbidity of motor activity in different follow-up periods. It can be seen from the results of VA-ECMO that the general trend is that with the extension of time on ECMO, the incidence of neonatal motor activity complications is increasing.

Meanwhile, the long-term prognosis in neonates undergoing ECMO is associated with the occurrence of short-term complications. According to the 2017 ESLO report, from 2013 to 2017, the CNS bleeding occurred in 12% of ECMO neonates who received respiratory support, and 39% of them survived. The CNS infarction occurred in 4% of neonates, and 33% of whom survived [31]. However, in contrast, for adult patients undergoing VA-ECMO, previous meta-analyses have estimated a cumulative rate of 13.3% for all reported neurological complications and 5.9-7.8% for ischemic and/or hemorrhagic stroke [32, 33].

Numerous factors influence neuropsychological complications in neonates undergoing ECMO. It is essential to determine how to collect detailed data before, during, and after ECMO for further analysis of neuropsychological complications in neonates. These detailed data should not only include the basic diseases, but also the feeding of newborns, intravenous administration of nutrients and nutrient components, drugs, and growth and development of neonates. Moreover, as a complex interplay among different factors associated with the underlying disease, pharmacological and non-pharmacological treatments and “iatrogenesis” are likely to determine a child’s neuropsychological outcome [34].

There are some strengths in this study. On the one hand, this review is a rigorous search, independent screening, as well as being the detailed reporting of methods and findings. On the other hand, this meta-analysis is one of the few studies that quantitatively analyzed the long-term neuropsychological complications associated with ECMO in neonates, which is more intuitive and clearer. In addition, due to data limitations, the long-term neuropsychological complications of neonatal CDH and neonates undergoing VA-ECMO were analyzed separately. Sensitivity analysis was used to analyze the heterogeneity of data, which could lay a foundation for the future research.

However, this study has several limitations. First, only observational studies were involved in the quantitative analysis. Second, the number of participants in each study who were included in the systematic review was uneven. Although the overall number was sufficient, the number of participants who could be included in each study was very small. Third, the included studies used a variety of outcome measures at different time points and could not be combined in the meta-analysis. Because the results of the reports that used the same test were different, the level of details was also different. As a number of the included studies did not provide more detailed data, it was impossible to extract the basic and effective data for analysis, which made it difficult to summarize the results and to identify the neuropsychological complications of a certain type of neonatal diseases. Children with severe congenital and acquired illness requiring prolonged hospitalization and intensive care likely suffer morbidity in the domains described, regardless of ECMO status. However, what cannot be gleaned from this study is the degree to which ECMO is a cause of these complications independently.

## Conclusions

This systematic review and meta-analysis of observational studies concentrated on the long-term follow-up of neuropsychological complications in neonates, despite methodological limitations of the included studies. The results showed that there were several neuropsychological complications during the long-term follow-up, and they might even affect children's quality of life. These results are of great significance in evaluating the neuropsychological development of neonates undergoing ECMO, and provide data basis for further research in the future. Certainly, additional RCTs with a larger sample size and a higher quality are needed to explore the causes of neuropsychological abnormalities and to guide us how to avoid such complications.

### Supplementary Information

**Additional file 1.** 

## Data Availability

All data generated or analysed during this study are included in this published article and its supplementary information files.

## Abbreviations

aEEG: Amplitude integrated EEG
CBCL: Child Behavior Checklist
CIs: Confidence intervals
ELSO: Extracorporeal life support organization
GRADE: Grading of Recommendations, Assessments, Developments and Evaluations
MABC: Movement Assessment Battery for Children
MOOSE: Meta-analysis of Observational Studies in Epidemiology
NOS: Newcastle-Ottawa Scale
PedsQL: Pediatric Quality of Life Inventory
PICOS: Population-Intervention-Comparison-Outcome-Study Design
PRISMA: Preferred Reporting Items for Systematic Reviews and Meta-analyses
RAKIT: Revised Amsterdam Intelligence Test
RAVLT: Rey Auditory Verbal Learning Test
RCFT: Rey Complex Figure Test
RCTs: Randomized controlled trials
RD: Risk difference
VA: Veno-Arterial
VV: Veno-Venous
WISC-III-NL: Wechsler Intelligence Scale for children
