# Supplementary material for: Long-term follow-up of neuropsychological complications in neonates undergoing extracorporeal membrane oxygenation: a systematic review and meta-analysis
Source: BMC Pediatr. 2024 Jan 24;24:77. doi: 10.1186/s12887-024-04564-x (PMC10807126; doi:10.1186/s12887-024-04564-x)
Supplement: Supplementary file 1 — Additional file 1. [file 12887_2024_4564_MOESM1_ESM.docx]

**Long-term follow-up of neuropsychological complications in neonates undergoing extracorporeal membrane oxygenation: a systematic review and meta-analysis**

**Systematic Review Search Strategy.**

**PubMed**

"extracorporeal membrane oxygenation"[MeSH Terms] OR (("extracorporeal"[tw] OR "extra-corporeal"[tw] OR "extra corporeal"[tw]) AND "membrane"[tw] AND "oxygenation"[tw]) OR "extracorporeal membrane oxygenation"[tw] OR "extra-corporeal membrane oxygenation"[tw] OR "extra corporeal membrane oxygenation"[tw] OR "ECMO"[tw] OR "extracorporeal oxygenation"[tw] OR "extra corporeal oxygenation"[tw] OR "extra-corporeal oxygenation"[tw] OR "extracorporeal life support"[tw] OR "extra corporeal life support"[tw] OR "extra-corporeal life support"[tw] OR "ECLS"[tw] OR "extrapulmonary oxygenation"[tw] OR "extracorporeal carbon dioxide removal"[tw] OR "extracorporeal membrane oxygenation device"[tw] OR ECPR[tw] OR "extracorporeal cardiopulmonary resuscitation"[tw]

**AND** ("neurocognitive"[tw] OR "neuro-cognitive"[tw] OR "neuro cognitive"[tw] OR "neurocognition"[tw] OR "neurodevelopmental"[tw] OR "neuro-developmental"[tw] OR "neuro developmental"[tw] OR "neurodevelopment"[tw] OR "neuro-development"[tw] OR "neuro development"[tw] OR "neurologic"[tw] OR "neurological"[tw] OR "cognitive"[tw] OR "brain injury"[tw] OR "brain injuries"[tw] OR "Seizures"[Mesh] OR "seizure"[tw] OR "seizures"[tw] OR "Quality of Life"[Mesh] OR "quality of life"[tw] OR "health-related quality of life"[tw] OR "HRQOL"[tw] OR "functional outcome"[tw] OR "functional outcomes"[tw])

**AND** ("neonate"[tw] OR "neo-nate"[tw] OR "neoborn"[tw] OR "neonatal"[tw] OR "neo-natal"[tw] OR "neonatality"[tw] OR "neonates"[tw] OR "neo-nates"[tw] OR "neonatals"[tw])

Searched 10/06/2022, limited to in the last 10 years. 181 results

**Web of Science**

TS=((("extracorporeal" OR "extra-corporeal" OR "extra corporeal") AND "membrane" AND "oxygenation") OR "extracorporeal membrane oxygenation" OR "extra-corporeal membrane oxygenation" OR "extra corporeal membrane oxygenation" OR "ECMO" OR "extracorporeal oxygenation" OR "extra-corporeal oxygenation" OR "extra corporeal oxygenation" OR "extracorporeal life support" OR "extra-corporeal life support" OR "extra corporeal life support" OR "ECLS" OR "extrapulmonary oxygenation" OR "extracorporeal carbon dioxide removal" OR "extracorporeal membrane oxygenation device" OR "ECPR" OR "extracorporeal cardiopulmonary resuscitation")

**AND** TS=(("neurocognitive" OR "neuro-cognitive" OR "neuro cognitive" OR "neurocognition" OR "neurodevelopmental" OR "neuro-developmental" OR "neuro developmental" OR "neurodevelopment" OR "neuro-development" OR "neuro development" OR "neurologic" OR "neurological" OR "cognitive" OR "brain injury" OR "brain injuries" OR "seizure" OR "seizures" OR "quality of life" OR "health-related quality of life" OR "HRQOL" OR "functional outcome" OR "functional outcomes"))

**AND** TS=(("neonate" OR "neo-nate" OR "neoborn" OR "neonatal" OR "neo-natal" OR "neonatality" OR "neonates" OR "neo-nates" OR "neonatals"))

Searched 10/06/2022, limited to in the last 10 years. 256 results

**Cochrane**

[mh "extracorporeal membrane oxygenation"] OR (("extracorporeal":ti,ab,kw OR "extra-corporeal":ti,ab,kw OR "extra corporeal":ti,ab,kw) AND ("membrane":ti,ab,kw AND "oxygenation":ti,ab,kw) OR "extracorporeal membrane oxygenation":ti,ab,kw OR "extra-corporeal membrane oxygenation":ti,ab,kw OR "extra corporeal membrane oxygenation":ti,ab,kw OR "ECMO":ti,ab,kw OR "extracorporeal oxygenation":ti,ab,kw OR "extra-corporeal oxygenation":ti,ab,kw OR "extra corporeal oxygenation":ti,ab,kw OR "extracorporeal life support":ti,ab,kw OR "extra-corporeal life support":ti,ab,kw OR "extra corporeal life support":ti,ab,kw OR "ECLS":ti,ab,kw OR "extrapulmonary oxygenation":ti,ab,kw OR "extracorporeal carbon dioxide removal":ti,ab,kw OR "extracorporeal membrane oxygenation device":ti,ab,kw OR "ECPR":ti,ab,kw OR "extracorporeal cardiopulmonary resuscitation":ti,ab,kw)

Outcomes:

[mh Seizures] OR [mh "Quality of Life"] OR ("neurocognitive":ti,ab,kw OR "neuro-cognitive":ti,ab,kw OR "neuro cognitive":ti,ab,kw OR "neurocognition":ti,ab,kw OR "neurodevelopmental":ti,ab,kw OR "neuro-developmental":ti,ab,kw OR "neuro developmental":ti,ab,kw OR "neurodevelopment":ti,ab,kw OR "neuro-development":ti,ab,kw OR "neuro development":ti,ab,kw OR "neurologic":ti,ab,kw OR "neurological":ti,ab,kw OR "cognitive":ti,ab,kw OR "brain injury":ti,ab,kw OR "brain injuries":ti,ab,kw OR "seizure":ti,ab,kw OR "seizures":ti,ab,kw OR "quality of life":ti,ab,kw OR "health-related quality of life":ti,ab,kw OR "HRQOL":ti,ab,kw OR "functional outcome":ti,ab,kw OR "functional outcomes":ti,ab,kw)

[mh neonate] OR ('neonate':ti,ab,kw OR 'neo-nate':ti,ab,kw OR 'neoborn':ti,ab,kw OR 'neonatal':ti,ab,kw OR 'neo-natal':ti,ab,kw OR 'neonatality':ti,ab,kw OR 'neonates':ti,ab,kw OR 'neo-nates':ti,ab,kw OR 'neonatals':ti,ab,kw)

Searched 10/06/2022, limited to in the last 10 years. 5 results

**EMBASE**

'extracorporeal' OR 'extra-corporeal' OR 'extra corporeal' AND 'membrane':ab,ti AND 'oxygenation' OR 'extracorporeal membrane oxygenation' OR 'extra-corporeal membrane oxygenation' OR 'extra corporeal membrane oxygenation' OR 'ecmo' OR 'extracorporeal oxygenation'/syn OR 'extracorporeal oxygenation' OR 'extra-corporeal oxygenation' OR 'extra corporeal oxygenation' OR 'extracorporeal life support' OR 'extra-corporeal life support' OR 'extra corporeal life support' OR 'ecls' OR 'extrapulmonary oxygenation' OR 'extracorporeal carbon dioxide removal' OR 'extracorporeal membrane oxygenation device'/syn OR 'ecpr' OR 'extracorporeal cardiopulmonary resuscitation' AND ('neurocognitive':de,ab,ti OR 'neuro-cognitive':de,ab,ti OR 'neuro cognitive':de,ab,ti OR 'neurocognition':de,ab,ti OR 'neurodevelopmental':de,ab,ti OR 'neuro-developmental':de,ab,ti OR 'neuro developmental':de,ab,ti OR 'neurodevelopment':de,ab,ti OR 'neuro-development':de,ab,ti OR 'neuro development':de,ab,ti OR 'neurologic':de,ab,ti OR 'neurological':de,ab,ti OR 'cognitive':de,ab,ti OR 'brain injury':de,ab,ti OR 'brain injuries':de,ab,ti OR 'seizure':de,ab,ti OR 'seizures':de,ab,ti OR 'quality of life':de,ab,ti OR 'health-related quality of life':de,ab,ti OR 'HRQOL':ab,ti OR 'functional outcome':ab,ti OR 'functional outcomes':ab,ti) AND ('neonate':de,ab,ti OR 'neo-nate':de,ab,ti OR 'neoborn':de,ab,ti OR 'neonatal':de,ab,ti OR 'neo-natal':de,ab,ti OR 'neonatality':de,ab,ti OR 'neonates':de,ab,ti OR 'neo-nates':de,ab,ti OR 'neonatals':de,ab,ti)

Searched 10/06/2022, limited to in the last 10 years. 208 results
